# Adhesion GPCRs in Kidney Development and Disease

**DOI:** 10.3389/fcell.2018.00009

**Published:** 2018-02-06

**Authors:** Salvador Cazorla-Vázquez, Felix B. Engel

**Affiliations:** Department of Nephropathology, Experimental Renal and Cardiovascular Research, Institute of Pathology, Friedrich-Alexander-Universität Erlangen-Nürnberg, Erlangen, Germany

**Keywords:** kidney, adhesion G protein-coupled receptor, renal cell carcinoma, chronic kidney disease, lupus nephritis, diabetic nephropathy

## Abstract

Chronic kidney disease (CKD) represents the fastest growing pathology worldwide with a prevalence of >10% in many countries. In addition, kidney cancer represents 5% of all new diagnosed cancers. As currently no effective therapies exist to restore kidney function after CKD- as well as cancer-induced renal damage, it is important to elucidate new regulators of kidney development and disease as new therapeutic targets. G protein-coupled receptors (GPCRs) represent the most successful class of pharmaceutical targets. In recent years adhesion GPCRs (aGPCRs), the second largest GPCR family, gained significant attention as they are present on almost all mammalian cells, are associated to a plethora of diseases and regulate important cellular processes. aGPCRs regulate for example cell polarity, mitotic spindle orientation, cell migration, and cell aggregation; all processes that play important roles in kidney development and/or disease. Moreover, polycystin-1, a major regulator of kidney development and disease, contains a GAIN domain, which is otherwise only found in aGPCRs. In this review, we assess the potential of aGPCRs as therapeutic targets for kidney disease. For this purpose we have summarized the available literature and analyzed data from the databases The Human Protein Atlas, EURExpress, Nephroseq, FireBrowse, cBioPortal for Cancer Genomics and the National Cancer Institute Genomic Data Commons data portal (NCIGDC). Our data indicate that most aGPCRs are expressed in different spatio-temporal patterns during kidney development and that altered aGPCR expression is associated with a variety of kidney diseases including CKD, diabetic nephropathy, lupus nephritis as well as renal cell carcinoma. We conclude that aGPCRs present a promising new class of therapeutic targets and/or might be useful as diagnostic markers in kidney disease.

## Kidney function

The main function of the kidney is to filter the blood while conserving water and maintaining the electrolyte homeostasis. A part of the filtrate is excreted (e.g., urea, ammonium, uric acid, metabolic waste, toxins) and another part is reabsorbed (e.g., solute-free water, sodium, bicarbonate, glucose, calcium, phosphate, and amino acids). In addition, the kidney has other functions such as gluconeogenesis, converting a precursor of vitamin D to its active form, and synthesizing the hormones erythropoietin and renin. This way the kidney regulates the volume of various body fluid compartments, fluid osmolality, acid-base balance, various electrolyte concentrations, removal of toxins and participates in the intermediary metabolism.

**Figure 1:**
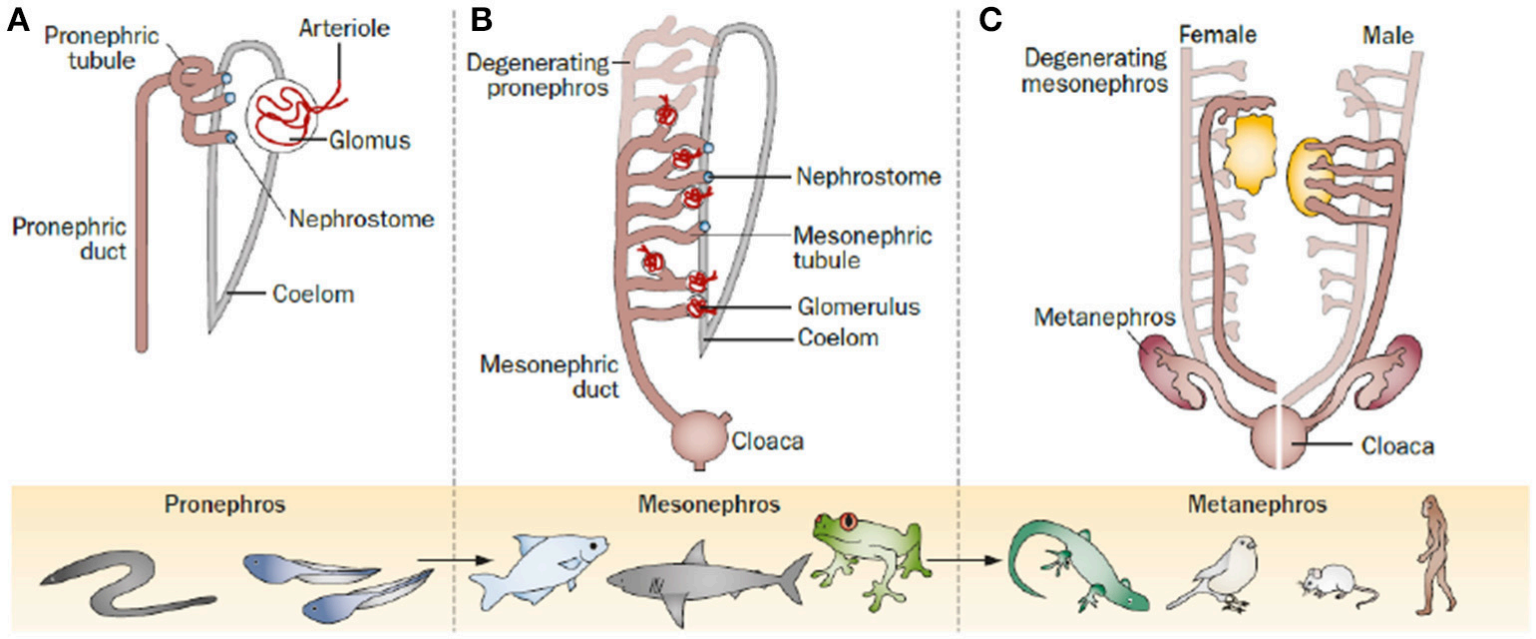
The kidney through evolution, as it proceeded through a series of successive phases, each marked by the development of a more advanced kidney: the pronephros, mesonephros, and metanephros. **(A)** The pronephros is the most immature form of kidney; it represents the first stage of kidney development in most animal species, but became functional only in ancient fish, such as lampreys or hagfish, or during the larval stage of amphibians. **(B)** The mesonephros represents the second stage of kidney development in most animal species, and represents the functional mature kidney in most fish and amphibians. It is made up of an increased number of nephrons, usually dozens to hundreds. **(C)** The metanephros represents the last stage of kidney development after degeneration of the pronephros and mesonephros in reptiles, birds and mammals, where it persists as the definitive adult kidney; it consists of a substantially increased number of nephrons, usually from thousands to millions. Romagnani et al. progenitors: an evolutionary conserved strategy for kidney regeneration. Reprinted by permission from Macmillan Publishers Ltd: (Romagnani et al., 2013).

The structural and functional unit of the kidney is the nephron which consists of a renal corpuscle and a renal tubule (Figure 1A; Romagnani et al., 2013). The renal corpuscle contains a tuft of capillaries (glomerulus) and the Bowman's capsule. The renal tubule extends from the capsule and has five anatomically and functionally different segments: the proximal tubule (proximal convoluted tubule followed by the proximal straight tubule), the loop of Henle (descending and ascending loop), the distal convoluted tubule, the connecting tubule, and the collecting duct. Blood filtration occurs in the glomerulus through two cell layers, fenestrated endothelial cells and podocytes, as well as their individual laminae and the basal membrane they both share. Resorption takes place via peritubular capillaries while the fluid flows from the Bowman's capsule down the tubule. The urine, final product of blood filtration, runs from the collecting ducts through the renal papillae, calyces, pelvis, and finally via the ureter into the urinary bladder.

## Kidney development

Due to different surrounding environments and thus different requirements on renal function, kidneys passed through different adaptive designs along evolution. Ultimately, this led to the intricate structures in the mammalian kidney. The kidney arises from the intermediate mesoderm and proceeds through three successive developmental phases representing different evolutionary patterns: pronephros, mesonephros, and metanephros (Figure 1; Saxén et al., 1986). The first evidence of kidney organogenesis is the formation of a primary nephric (pronephric) duct. As it grows caudally, a linear array of epithelial tubules forms and extends medioventrally giving rise to an anterior rudimentary pronephros, in which the tubules are not yet directly linked with the capillary tuft (Romagnani et al., 2013). The pronephros (Figure 1A) is active in embryos of fish and larvae of amphibians as well as in some adult primitive fish. In more advanced vertebrates, the pronephros is frequently nonfunctional and soon replaced by the mesonephros. The anterior part of the pronephric duct degenerates while the caudal portion persist and serves as the central component of the excretory system (mesonephric or Wolffian duct). The middle portion induces another set of tubules, the mesonephros (Figure 1B), which is in mammals the source of hematopoietic stem cells. Subsequently, the mesonephros either regresses or becomes sperm-carrying tubes (epididymis, vas deferens and seminal vesicles) in male individuals. In amphibians and most fish the mesonephros represents the permanent kidney, while in mammals, birds, and reptiles the mesonephros regresses and the metanephros is formed.

**Figure 2:**
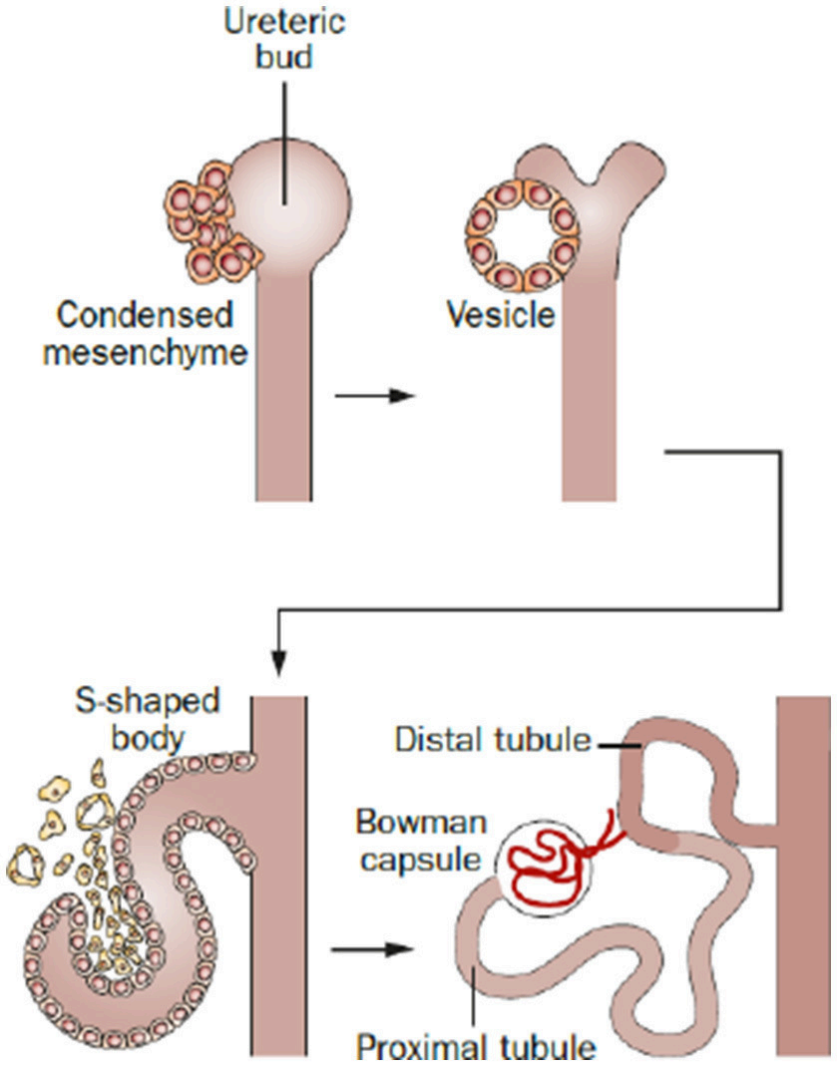
Phases of nephron development in all animals. The metanephric mesenchyme condenses around the ureteric bud and is induced to convert into epithelium and generates in sequence a vesicle and an S-shaped body. Then, the S-shaped body becomes invaded by blood vessels at one extremity and elongates and segmentates at the other, thus generating the whole nephron. This sequence of events is similar during development across all animal species. Reprinted by permission from Macmillan Publishers Ltd: (Romagnani et al., 2013).

The metanephros (Figure 1C) is the permanent kidney in reptiles, birds, and mammals. It develops from a caudal diverticulum of the Wolffian duct, which is induced to branch by signals from the surrounding metanephric mesenchyme. This epithelial branch is called ureteric bud (Schmidt-Ott et al., 2005; Dressler, 2008). The ureteric bud epithelia proliferate, migrate, and gradually invade the metanephric mesenchyme as it undergoes branching morphogenesis (Watanabe and Costantini, 2004), which is mediated by glial-derived neurotrophic factor (Gdnf). Gdnf is released by metanephric mesenchymal cells and binds to the Ret receptor on epithelial ureteric bud cells inducing migration, invasion and branching (Towers et al., 1998; Costantini, 2010). In the end, the ureteric bud branches from the collecting system of the kidney (bladder trigone, ureters, renal pelvis, and collecting ducts), which is controlled by multiple patterning factors such as c-Ret, Spry1, ETv5, Robo2, Sox9, Wnt11, Angiotensin II, Fgfr2, Bmp4, At1r, FoxC2, and Pax2 (Yosypiv, 2008; Yosypiv et al., 2008; Reidy and Rosenblum, 2009; Faa et al., 2012). Surrounding the tips of the ureteric bud, metanephric mesenchymal cells condensate and aggregate into the so-called cap mesenchyme, a process mainly driven by Pax2 and Wnt4 (Sariola, 2002; Figure 2). Afterwards, the cap mesenchyme progressively undergoes mesenchymal-to-epithelial transition (MET) forming the renal vesicles (Carroll et al., 2005) via Wnt4, Lhx1, and Fgf8 signaling and downregulation of Six2 and Cited2 expression (Kispert et al., 1998; Davies, 1999; Brunskill et al., 2008). Subsequently, the vesicles undergo segmentation patterning followed by fusion with the ureteric branches to form a uriniferous tubule (Georgas et al., 2009). During the following development, renal vesicles change their appearance and proceed through the stages comma-shaped body, S-shaped body and capillary loop stage (Little et al., 2007). During these stages, markers for proximal and distal tubules of the future nephron start to be expressed, and the primitive glomeruli evolve by incorporating vascular loops and allowing endothelial cells to get in contact with visceral epithelial cells (early podocytes) forming the glomerular filtration barrier. Transcription factors such as Wt1 and Pod1 play a central role during this process of morphogenesis (Rico et al., 2005). Interstitial cells and vasculature are formed from a multipotent pool of progenitor cells in the metanephric mesenchyme (Kispert et al., 1998).

## Resemblance of adhesion G protein-coupled receptors (aGPCRs) and polycystin-1

**Figure 3:**
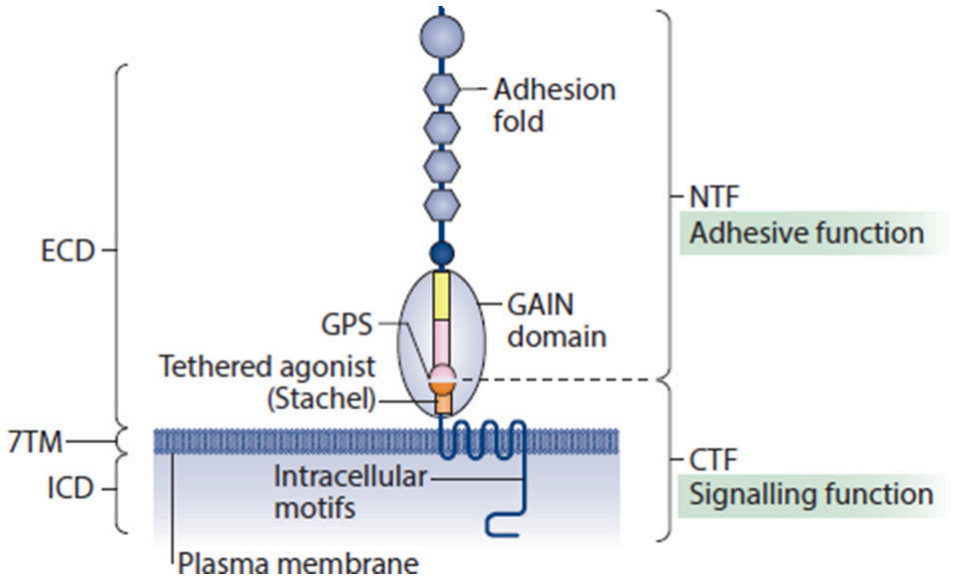
Adhesion G protein-coupled receptors possess structural elements of adhesion molecules and GPCRs. Their extended extracellular domain (ECD) usually contains a collection of adhesion motifs that can engage with cellular and matricellular interaction partners, and a juxtamembrane GPCR autoproteolysis-inducing (GAIN) domain, which is present in all aGPCRs. GAIN subdomain A (yellow rectangle), GAIN subdomain B (pink rectangle) and the GPCR proteolysis site (GPS) motif (pink and orange semicircles) are shown. The GAIN domain is directly connected to the seven-transmembrane (7TM) unit through a linker sequence of approximately 20 amino acids, known as the Stachel (stalk). Recently, this structural component of aGPCRs was identified as a tethered agonist, which stimulates metabotropic activity of several aGPCR homologs. Similar to the ECD, the intracellular domains (ICDs) of aGPCRs can be unusually large. It is estimated that more than one-half of all known aGPCRs undergo auto-proteolytic cleavage that is catalyzed through the GAIN domain, which is present on the cell surface as a non-covalent heterodimer between an amino-terminal fragment (NTF) and a carboxy-terminal fragment (CTF). The cleavage occurs at the evolutionarily highly conserved GPS. Reprinted by permission from Macmillan Publishers Ltd: (Langenhan et al., 2016).

aGPCRs represent one of five families of the GPCR superfamily, the largest group of membrane receptors in eukaryotes. In humans, the aGPCR family contains 33 members which can be subdivided into 9 subfamilies (I to IX) (Hamann et al., 2015). They are characterized by a long extracellular domain which comprises several adhesion domains, a 7 transmembrane domain and an intracellular domain. The extended N-terminus can be cleaved in almost all aGPCRs at the so-called GPCR proteolysis site (GPS) within the GPCR autoproteolysis-inducing (GAIN) domain (Figure 3). This property permits a variety of signaling modalities including activation of CTF-dependent or -independent signals via agonist interactions at the NTF or the CTF. In addition, NTF can bind either to partners at the surface of a neighboring cell or the extracellular matrix. Yet, the NTF can also be shed off to induce non-cell-autonomous signals in neighboring or in distant cells (Langenhan et al., 2013; Patra et al., 2013, 2014). However, some aGPCRs containing a GPS motif do not undergo cleavage [e.g., ADGRC1 (CELSR1), ADGRF4 (GPR115), ADGRF2 (GPR111)]. ADGRA1 (GPR123) is the only aGPCR that does not contain a GAIN domain (Hamann et al., 2015).

GPCRs are of great clinical interest as they play an important role in the development of pharmacological-based therapies (Lagerström and Schiöth, 2008; Nieto Gutierrez and McDonald, 2017). In addition, aGPCRs are widely expressed (Hamann et al., 2015), play critical roles in many developmental processes (Luo et al., 2011; Geng et al., 2013; Ludwig et al., 2016; Musa et al., 2016; Sigoillot et al., 2016), and are also involved in a variety of diseases. For example, aGPCRs are involved in the control of innate effector functions and the susceptibility for and onset of (auto)inflammatory conditions (Lin et al., 2017), in tumorigenesis (Aust et al., 2016), and pulmonary disease (Ludwig et al., 2016), and they are linked to psychiatric disorders (Lanoue et al., 2013). Yet, little is known about the downstream signaling mechanisms of aGPCRs. In recent years, however, evidence has been provided that aGPCRs can control cell polarity (Strutt et al., 2016), mitotic spindle orientation (Müller et al., 2015), cell migration (Valtcheva et al., 2013; Strutt et al., 2016), and cell aggregation (Hsiao et al., 2011) and that they serve as mechanosensors (Scholz et al., 2016). These are all processes that also play important roles in kidney development and disturbance of these processes are known to contribute to kidney disease (Sariola, 2002; Schordan et al., 2011; Cabral and Garvin, 2014; Xia et al., 2015; Gao et al., 2017; Kunimoto et al., 2017). Thus, we wondered whether also aGPCRs might play a role in renal physiology and pathophysiology to determine their potential as therapeutic targets for kidney disease.

As detailed below, the importance of aGPCRs in kidney development is underlined by initial reports in the literature. Moreover, it is intriguing that the 11 transmembrane domain receptor polycystin-1, a major regulator of kidney development and disease, is the only GAIN-domain containing protein known so far that does not belong to the aGPCR family (Cai et al., 2014). Polycystin-1 plays a role in renal tubular development and functions in a complex with polycystin-2 as a fluid shear sensor. Mutations in the corresponding genes, *Pkd1* and *Pkd2*, have been associated with autosomal dominant polycystic kidney disease (ADPKD) (Ferreira et al., 2015). Furthermore, it has been shown that autoproteolytic cleavage at a GPS motif is required for polycystin-1 trafficking to cilia and that only a cleavable form of polycystin-1 can rescue the embryonically lethal *Pkd1*-null mutation in mice (Cai et al., 2014).

## Search strategy

In order to determine what has so far been made publically available in regards to the role of aGPCRs in kidney development and disease, we performed a literature search using Pubmed (https://www.ncbi.nlm.nih.gov/pubmed/). Search terms included “kidney” or “renal,” “development,” “disease” and/or the official or alternative individual names of every aGPCR. In addition, we cross-checked our literature search results with the literature information available in the IUPHAR/BPS database (http://www.guidetopharmacology.org/). Moreover, we performed a comprehensive search utilizing Google Scholar (https://scholar.google.de/), Google (https://www.google.de/) and Google images (https://images.google.de/). Finally, we analyzed the following databases:

The Human Protein Atlas (https://www.proteinatlas.org/). The Human Protein Atlas (HPA) is a Swedish-based program that aims “to map all the human proteins in cells, tissues and organs using integration of various Omics technologies, including antibody-based imaging, mass spectrometry-based proteomics, transcriptomics and systems biology” (Uhlén et al., 2015; Uhlen et al., 2017; Thul et al., 2017). For our review we utilized the “Tissue Atlas” that shows the distribution of proteins across all major tissues and organs in the human body including the kidney. The provided data are based on RNAseq and immunohistochemistry on tissue microarrays revealing spatial distribution, cell type specificity and the rough relative abundance of proteins in these tissues.

EURExpress (http://www.eurexpress.org/ee/) (Diez-Roux et al., 2011). The EURExpress database provides RNA expression patterns by means of *in situ* hybridization on paraffin-embedded sections, gelatin-embedded sections and cryosections from E14.5 wild type murine embryos. For some genes, data are also available for other embryonic stages. Note, that this database is not fully annotated, fails to indicate in several cases organs exhibiting a positive expression signal, and does not always provide the necessary resolution to associate the expression pattern with a distinct cell type.

Nephroseq (https://www.nephroseq.org/resource/login.html) (November 2017, University of Michigan, Ann Arbor, MI). Nephroseq is a platform that allows integrative data mining of publicly available renal developmental (human) as well as renal disease (model as well as clinical) gene expression data sets which are gathered and curated by an experienced team of data scientists, bioinformaticians, and nephrologists. Thus, it allows identifying genes associated with known disease phenotypes to obtain a better understanding of its role in renal physiology and pathophysiology. In regards to healthy adult kidneys it provides microarray-based information from 5 samples regarding the enrichment of a gene in one of the following compartments: glomeruli, inner renal cortex, inner renal medulla, outer renal cortex, outer renal medulla, papillary tips, and renal pelvis (Higgins et al., 2004). Here, we considered genes as significantly regulated if the *p*-value was <0.05.

FireBrowse (http://firebrowse.org/). FireBrowse is a platform that enables an efficient analysis of a possible association of a candidate gene with cancer based on DNA and RNA sequence data. The platform accesses Broad Institute's TCGA (The Cancer Genome Atlas) GDAC (Genome Data Analysis Center) Firehose database, “one of the deepest and most integratively characterized open cancer datasets in the world” (over 80,000 sample aliquots from >11,000 cancer patients, spanning 38 unique disease cohorts). Here, we highlight information on >2.5-fold change of RNA expression. Note, no *p*-values are available.

cBioPortal for Cancer Genomics (http://www.cbioportal.org/) (Cerami et al., 2012; Gao et al., 2013). cBioPortal (cBio) is another platform based on TCGA to associate candidate genes with cancer based on data from 168 cancer studies. It comprises DNA copy-number data, mRNA and microRNA expression data, non-synonymous mutations, protein-level and phosphoprotein level (RPPA) data, DNA methylation data, and limited de-identified clinical data.

National Cancer Institute Genomic Data Commons data portal (NCIGDC) (https://portal.gdc.cancer.gov/). Similar to the two databases above, NCIGDC allows based on TCGA the association of candidate genes with cancer based on 32,555 cases. Data are provided for 22,144 genes including 3,115,606 mutations.

Our analysis revealed that the majority of the 33 human aGPCRs and several orthologues have been in some aspect related to kidney development or disease (Tables 1, 2) as detailed below.

**Table 1:** Adhesion GPCRs associated to kidney development.

**aGPCR**	**References**	**Database**	**Expression**	**Spatio-temporal information**	**Gain/loss of function**	**Function/phenotype**
*Adgrl1*	Masiero et al., 2013	HPA	IHC, NB	adult (human: tubules)	-	-
*Adgrl2*	Sugita et al., 1998; Ichtchenko et al., 1999; Masiero et al., 2013	-	NB	adult (human, rat)	-	-
*Adgrl3*	-	HPA, NS	IHC, microarray	adult (human: tubules, glomerulus)	-	-
*Adgrl4*	Nie et al., 2012	EX, HPA	IF, IHC, ISH, reporter line	embryonic (E10.5 mouse: vessels; E14.5 mouse), adult (human vessels)	KO and double KO mouse (with *Adgrf5*)	glomerular thrombotic angiopathy
*Adgre1*	-	NS	microarray	adult (human: glomerulus)	-	-
*Adgre2*	-	-	-	-	-	-
*Adgre3*		HPA	IHC	adult (human: tubular cells)	-	-
*Adgre4*	-	-	-	-	-	-
*Adgre5*	Hamann et al., 1996; Qian et al., 1999; de la Lastra et al., 2003; Formstone et al., 2010; Toomey et al., 2014	HPA, NS	IF, IHC, NB, RT-PCR, microarray, reporter line	embryonic (E14.5 mouse: collecting system), juvenile (4-month-old mouse: podocytes, mesangial cells), adult (zebrafish/ pig/ human: glomerulus, pelvis/ mouse: mesangial cells)	KO mouse	-
*Adgra1*	Formstone et al., 2010	-	RT-PCR	adult (zebrafish)	-	-
*Adgra2*	Huang et al., 2008	EX, NS	ISH, RT-PCR, microarray	embryonic (E14.5 mouse), adult (rat/ human: pelvis)	-	-
*Adgra3*	-	EX, HPA	IHC, ISH	embryonic (E14.5 mouse: nephrogenic cortex), adult (human: tubular cells)	-	-
*Adgrc1*	Formstone et al., 2010; Yates et al., 2010a; Brzoska et al., 2016	EX, HPA	IF, IHC, ISH, RT-PCR	embryonic (E14.5 mouse, E18.5 mouse: podocytes, proximal tubule, collecting duct stalks, S-shaped body); adult (zebrafish/ human: vessels)	Mice carrying a loss of function mutation	ureteric bud branching
*Adgrc2*	-	EX, HPA, NS	IHC, ISH, microarray	embryonic (E14.5 mouse), adult (human: medulla)	-	-
*Adgrc3*	-	-	-	-	-	-
*Adgrd1*	-	-	-	-	-	-
*Adgrd2*	-	-	-	-	-	-
*Adgrf1*	Alva et al., 2006; Prömel et al., 2012	HPA, NS	IHC, RT-PCR, microarray, reporter line	adult (human: glomerulus, papillary tips)	KO mouse	-
*Adgrf2*	-	-	-	-	-	-
*Adgrf3*	-	NS	microarray	adult (human: glomerulus)	-	-
*Adgrf4*	Alva et al., 2006	-	RT-PCR, reporter line	-	KO mouse	-
*Adgrf5*	Alva et al., 2006; Nie et al., 2012	NS	IF, RT-PCR, microarray	embryonic (E12.5 mouse: vessels), adult (human: cortex, medulla, papillary tips/ mouse: glomerulus)	KO and double KO mouse (with *Adgrl4*)	glomerular thrombotic angiopathy
*Adgrb1*	Calderón-Zamora et al., 2017	-	IHC, RT-PCR	adult (human)	-	-
*Adgrb2*	-	HPA, NS	IHC, microarray	adult (human: tubules, pelvis)	-	-
*Adgrb3*	-	NS	microarray	adult (human: glomerulus)	-	-
*Adgrg1*	Matsushita et al., 1999; Shashidhar et al., 2005	EX, NS	ISH, NB, WB, microarray	embryonic (E12 mouse: ureteric branches), adult (human: cortex)	-	-
*Adgrg2*	-	-	-	-	-	-
*Adgrg3*	Formstone et al., 2010	-	RT-PCR	adult (zebrafish)	-	-
*Adgrg4*	-	NS	microarray	adult (human: glomerulus)	-	-
*Adgrg5*	-	HPA	IHC	adult (human: tubular cells)	-	-
*Adgrg6*	Formstone et al., 2010; Karner et al., 2015	EX, HPA, NS	IHC, ISH, RT-PCR, microarray	embryonic (E14.5 mouse: collecting system), adult (human: pelvis, vessels/ mouse: cortical collecting duct/ zebrafish)	-	-
*Adgrg7*	Formstone et al., 2010	-	RT-PCR	adult (zebrafish)	-	-
*Adgrv1*	-	HPA, NS	IHC, microarray	adult (human: medulla)	-	-

*EX, EURExpress; HPA, The Human Protein Atlas; NS, Nephroseq; IF, immunofluorescence; IHC, immunohistochemistry; ISH, in situ hybridization; NB, Northern blot; RT-PCR, real-time PCR; WB, Western blot*.

**Table 2:** Adhesion GPCRs associated to kidney disease.

**aGPCR member**	**References**	**Nephroseq (x-fold)**	**FireBrowse (x-fold)**			**NCIGDC**	**cBio**
			**ccRCC**	**chRCC**	**pRCC**		
*Adgrl1*	-	mDN (1.596)	0.420	1.870	0.919	-	-
*Adgrl2*	-	IgA (1.734), DN (1.791), LN (2.228) CKD (2.204)	1.060	0.867	0.766	-	-
*Adgrl3*	-	-	0.590	0.124	0.076	-	-
*Adgrl4*	Nie et al., 2012	IgA (2.757), LN (2.493), mDN (1.525)	2.130	0.405	0.179	-	chRCC (11%)
*Adgre1*	-	DN (1.566), mDN (1.654), mLN (3.238)	7.740	1.95	3.210	-	-
*Adgre2*	-	V (1.718), LN (1.756)	5.750	1.110	2.270	-	-
*Adgre3*	-	CKD (5.716)	1.920	0.518	2.510	-	-
*Adgre4*	-	CKD (6.363)	-	-	-	-	-
*Adgre5*	-	IgA (2.243), DN (2.488), V (2.278) HT (2.132), FSGS (2.121) MCD (1.642), LN (2.264) MG (1.645)	2.550	0.336	1.960	-	-
*Adgra1*	-	CKD (5.460), mDN (3.564)	0.081	1.390	0.138	-	-
*Adgra2*	-	APOL1 (1.526)	2.920	0.754	0.219	-	-
*Adgra3*	-	CKD (2.253), APOL1 (1.905)	0.857	0.476	1.840	-	chRCC (15%)
*Adgrc1*	-	-	1.090	0.176	0.871	ccRCC (11%) pRCC (10%)	ccRCC (15%)
*Adgrc2*	-	-	0.577	0.199	0.709	-	-
*Adgrc3*	-	-	2.190	3.060	4.770	ccRCC (12%) pRCC (10%)	chRCC (15%)
*Adgrd1*	-	mDN (1.630)	0.288	0.073	0.325	-	-
*Adgrd2*	-	CKD (2.294)	1.220	-	1.870	-	-
*Adgrf1*	-	mDN (2.708), CKD (5.413)	0.002	0.544	0.146	-	-
*Adgrf2*	-	CKD (6.115)	0.966	0.084	2.560	-	-
*Adgrf3*	-	-	0.050	0.160	0.099	-	-
*Adgrf4*	-	CKD (5.402)	2.150	0.118	3.310	-	chRCC (11%)
*Adgrf5*	Nie et al., 2012	-	0.702	1.600	0.056	-	-
*Adgrb1*	Calderón-Zamora et al., 2017; Mathema and Na-Bangchang, 2017	-	0.910	0.010	1.040	-	chRCC (11%)
*Adgrb2*	-	CKD (1.786)	0.424	0.355	2.110	-	chRCC (14%)
*Adgrb3*	-	CKD (3.092)	0.169	0.052	0.240	-	-
*Adgrg1*	-	FSGS (3.100), CKD (2.189), mDN (2.145)	0.703	3.890	0.476	-	-
*Adgrg2*	Kudo et al., 2007	DN (1.701), CKD (4.762), mDN (2.221), mLN (2.832)	2.340	1.180	0.413	-	chRCC (11%)
*Adgrg3*	Wang et al., 2013	mDN (2.938)	2.230	3.890	1.610	-	-
*Adgrg4*	-	CKD (4.004)	1.160	0.539	1.490	-	-
*Adgrg5*	-	CKD (1.605)	2.410	0.941	2.100	-	-
*Adgrg6*	-	DN (1.942), CKD (3.872), mLN (1.700)	1.900	0.418	2.00	-	-
*Adgrg7*	-	CKD (5.656)	1.990	2.080	1.970	-	-
*Adgrv1*	-	CKD (6.362)	0.072	0.399	0.028	ccRCC (16%) pRCC (19%)	chRCC (11%)

*APOL1, APOL1-associated kidney disease; ccRCC, clear cell renal cell carcinoma; chRCC, chromophobe renal cell carcinoma; CKD, chronic kidney disease; DN, diabetic nephropathy; FSGS, focal segmental glomerulosclerosis; HT, hypertension-injured kidney; IgA, IgA nephropathy; LN, lupus nephritis; MCD, minimal change disease; mDN, mouse model of diabetic nephropathy; MG, membranous glomerulopathy; mLN, mouse Berthier model of lupus nephritis with proteinuria; pRCC, papillary renal cell carcinoma; V, vasculitis-injured kidney; red boxes, <2.5-fold downregulation; green boxes, >2.5-fold upregulation*.

## aGPCRs in kidney development

### *Adgrc1* (*Celsr1*) is required for ureteric bud branching

The best characterized aGPCR in regards to its function during kidney development is *Adgrc1* (*Celsr1*). Yates and co-workers demonstrated in 2010 that *Adgrc1* and *Vangl2* are required for normal lung branching morphogenesis (Yates et al., 2010b). In the same year they reported furthermore that *Vangl2* is required for mammalian kidney-branching morphogenesis (Yates et al., 2010a). In this manuscript they also described Adgrc1 expression in podocytes, proximal tubular cells, collecting duct stalks and S-shaped bodies in the nephrogenic cortex of E18.5 mouse embryos. The study was based on an antibody whose specificity was validated by utilizing loss-of-function *Celsr1*^*Crsh*/*Crsh*^ embryos (Yates et al., 2010b). Yet, the authors did not analyze the role of *Adgrc1* during kidney development. Data from the EURExpress database (Diez-Roux et al., 2011), released in 2011, suggest that *Adgrc1* is expressed at E14.5 in mice and further indicated that *Adgrc1* plays a role in kidney development. Finally, Bróska and co-workers demonstrated in 2016 that *Adgrc1* is required, in association with the planar cell polarity gene *Vangl2*, for ureteric bud branching (Brzoska et al., 2016). Mice carrying mutations in *Adgrc1* (*Celsr1*^*Crsh*/*Crsh*^ and *Celsr1*^*Crsh*/+^*)* or *Vangl2* (*Vangl2*^*Lp*/+^) exhibited branching defects in the ureteric tree at E13.5, which were more severe in *Celsr1*^*Crsh*/+^*:Vangl2*^*Lp*/+^ mice. At E17.5 kidneys exhibited overall growth retardation (*Celsr1*^*Crsh*/+^*:Vangl2*^*Lp*/+^ > *Celsr1*^*Crsh*/*Crsh*^ > *Celsr1*^*Crsh*/+^) and histological analysis revealed mitotic spindle misorientation (along the longitudinal axis of the kidney tubules), dilation of cortical tubules in *Celsr1*^*Crsh*/*Crsh*^ mutants and an up-regulation of *Gdnf* and *Ret* mRNA levels in the *Celsr1*^*Crsh*/+^*:Vangl2*^*Lp*/+^ mutants. It is noteworthy that cleavage of Adgrc1 at its GPS motif has so far not been reported (Formstone et al., 2010; Hamann et al., 2015). Thus, GPS-based cleavage of Adgrc1 seems not to play a role in kidney development.

During adult renal physiology, *Adgrc1* seems not to play a major role as none of the databases indicates its expression in adult renal cells. In adult zebrafish, a low expression of the *Adgrc1* homolog *adgrc1a* (*celsr1a*) has been described in the kidney compared to the expression level in whole embryos at 10 h post fertilization (hpf) based on quantitative real-time PCR (Harty et al., 2015). However, considering only the expression levels in the investigated adult zebrafish organs (6 months), *adgrc1a* is markedly enriched in kidney, intestine, and skin compared to brain, eye, heart, liver, skeletal muscle, ovaries and testis (Harty et al., 2015). The only information available in the utilized developmental databases is that ADGRC1 is expressed in renal vessels (HPA database).

### aGPCRs strongly correlated to renal development

In this section, we discuss aGPCRs whose correlation to kidney development is strongly supported by data of several databases.

#### Adgrg6 (Gpr126)

Plays an essential role in neural, cardiac and ear development (Patra et al., 2014) as well as cartilage biology and spinal column development (Karner et al., 2015). The exact role and underlying signaling mechanism is still poorly understood. While no publication described so far the expression of *Adgrg6* during kidney development, *in situ* hybridization data available from the EURExpress database suggest that *Adgrg6* is expressed in E14.5 mouse embryos in the outgrowing ureteric bud branches of the metanephros (sections 9–12, 18–21). A possible renal function of *Adgrg6* is further supported by its RNA expression in the adult kidney of zebrafish (Harty et al., 2015) and humans (HPA database, vessels). Harty and coworkers revealed that *adgrg6* (*gpr126*) expression in the adult zebrafish kidney is comparable to the maximal expression level observed during development (3 days post fertilization, 3 dpf). *adgrg6* was only higher expressed in adult skeletal muscle (around 4 times enriched). Moreover, data from the Nephroseq database confirm the *ADGRG6* expression in the human kidney suggesting that it is enriched in the renal pelvis, which arises from the outgrowing ureteric bud branches. Similarly, Pradervand and coworkers published that *Adgrg6* is enriched in the cortical portion of the collecting duct in mice compared to the expression in the distal convoluted tubule and the connecting tubule based on microarray-based gene expression profiles (Pradervand et al., 2010). Collectively, the available data for *Adgrg6* suggest that it is expressed during kidney development as well as adulthood, being enriched in collecting duct cells. However, the described data are all RNA-based data as currently no reliable protein expression data are available. Moreover, no kidney phenotype has so far been described in *Adgrg6* knockout mice, neither in the lines that are embryonic lethal at E11.5 (Patra et al., 2013) nor in Taconic knockouts of which some are born alive (Monk et al., 2011). Currently, we are utilizing our model systems (Patra et al., 2013) including conditional mouse lines (Mogha et al., 2013) to determine if the role of *Adgrg6* might be underestimated in kidney development.

#### Adgre5 (CD97)

Is an activation-associated leukocyte antigen suggested to act as an adhesion molecule (Hamann et al., 2000). The association of Adgre5 with the decay accelerating factor (DAF) prevents cells from complement-mediated attack and therefore it is of great importance in the study of autoimmunity and graft rejection (Toomey et al., 2014). Already in 1999 first evidence, based on Northern blot analysis, was provided that *Adgre5* is expressed in the adult mouse kidney (Qian et al., 1999). A possible role of *Adgre5* in adult kidney physiology was further supported by the detection of *Adgre5* RNA in adult porcine kidney (de la Lastra et al., 2003), strong expression in adult zebrafish kidney [homolog *adgre5a* (*cd97a*)] (Harty et al., 2015), and more importantly by the detection of Adgre5 protein in intraglomerular mesangial cells of the adult mouse kidney (Jaspars et al., 2001) utilizing validated monoclonal antibodies (Hamann et al., 1996). In 2008, the analysis of a LacZ knockin reporter mouse line in combination with desmin expression analysis demonstrated that *Adgre5* is expressed in podocytes and mesangial cells of 4 months-old mice (Veninga et al., 2008). This is in agreement with data from the Nephroseq database that indicates that *ADGRE5* is enriched in the adult human glomerulus and renal pelvis. Yet, also despite a strong expression signal for *Adgre5* in the collecting duct system of E14.5 mouse embryos (EURExpress, sections 9–11, 18–21), 4 months-old *Adgre5* knockout mice showed no overt phenotype in the analyzed organs, including the kidney.

#### Subfamily VI

First evidence for a role of these family members during kidney physiology was provided by Abe and coworkers in 1999. They demonstrated by Northern blot analysis that ***Adgrf5*** (*Gpr116*/*Ig-Hepta*) is at low levels expressed in the adult rat kidney and identified by immunohistochemistry intercalated cells of the cortical collecting duct as the origin of expression (Abe et al., 1999). Previously, it has been demonstrated that it has a well-established role in lung surfactant homeostasis and that its deletion results in glucose intolerance and insulin resistance as well as a subtle vascular phenotype (Nie et al., 2012; Bridges et al., 2013; Fukuzawa et al., 2013; Yang et al., 2013; Niaudet et al., 2015). According to the Nephroseq database, *ADGRF5* is also expressed in the adult human kidney. Recently, transgenic mice expressing mCherry under the control of the *Adgrf5* promoter (*Gpr116*-mCherry) as well as knockout mice were generated (Lu et al., 2017). The analysis of 8 weeks-old reporter mice revealed that *Adgrf5* is expressed in endothelial cells of the microvessels in different organs. In the kidney, *Adgrf5* expression was also detected in microvessels and in glomeruli exclusively in endothelial cells and not in mesangial cells or podocytes. The previously described expression in intercalated cells of the cortical collecting duct was not confirmed. While *Adgrf5* knockout mice exhibited no obvious renal phenotype, *Adgrf5*/***Adgrl4*** (***Eltd1***) double knockout mice exhibited a hemolysis phenotype (Lu et al., 2017). In addition, these mice exhibited proteinuria as well as uremia indicating kidney failure. This was correlated to increased mesangial matrix and a damaged glomerular filtration barrier including loss of endothelial fenestration and fusion of podocytes foot processes. Minor albuminuria was already detected at birth. The authors concluded that *Adgrf5*/*Adgrl4* double knockout mice exhibit glomerular thrombotic microangiopathy. Note, *Adgrl4* knockout mice exhibited no obvious kidney phenotype. Moreover, endothelial-specific deletion of *Adgrf5*/*Adgrl4* utilizing *VE-Cad-Cre* mice, which are known to induce recombination in the endothelium of arteries and capillaries of the glomerulus of adult kidneys (Alva et al., 2006), resulted in no renal phenotype.

Prömel and coworkers investigated in 2012 by RT-PCR the expression of most members of subfamily VI [***Adgrf1*** (***Gpr110***), ***Adgrf2*** (***Gpr111***), ***Adgrf4*** (***Gpr115***), and *Adgrf5*]. They observed based on this analysis that *Adgrf1* is strongly expressed in the adult mouse kidney (Prömel et al., 2012), confirming previous data by Lum and coworkers demonstrating *ADGRF1* expression in the adult human kidney (Lum et al., 2010). In addition they detected very weak expression levels of *Adgrf4* (abundantly expressed in the skin) and *Adgrf5* (most abundantly expressed in lung, liver, and heart). No or at best faint expression was detected for *Adgrf2*. Subsequently, they generated reporter mouse lines and knockout mice for loss-of-function studies for *Adgrf1, Adgrf2*, and *Adgrf4*. The analysis of these mouse lines revealed strong expression of *Adgrf1* in the adult renal pelvis and ureter. For *Adgrf2* and *Adgrf4* no conclusive data were provided. Despite some indications of renal expression, the authors detected in none of the generated knockout mouse lines an obvious renal phenotype. According to the Nephroseq database, *ADGRF1* is enriched in the papillary tips, ***ADGRF3*** (***GPR113***) in the glomerulus, and *ADGRF5* in all renal structures, except for the pelvis, of the adult human kidney.

### aGPCRs weakly correlated to renal development

In this section, we discuss aGPCRs for which data are available but not sufficient, or even contradictory, to draw a strong conclusion in regards to a kidney-specific function.

#### Subfamily I

Like *Adgrf5, Adgrl4* is expressed in endothelial cells (Lu et al., 2017). Initially, it was described as a receptor upregulated in the adult heart (Nechiporuk et al., 2001). Subsequently, it has been shown to counteract pressure overload-induced myocardial hypertrophy (Xiao et al., 2012) and to regulate tumor angiogenesis (Masiero et al., 2013). During early mouse development, *Adgrl4* is detected in the heart endocardium, different regions of the brain and in the developing metanephros of E14.5 mice, according to EURExpress (sections 5–8, 14–17). The analysis of reporter mice revealed, similarly to *Adgrf5*, that *Adgrl4* is expressed in the adult microvasculature and in the kidney exclusively in endothelial cells and not in mesangial cells or podocytes of the glomeruli. In contrast to *Adgrl4*, little information is available for the other subfamily members. Based on Northern blot analysis it has been suggested that ***ADGRL1*** (***LPHN1***) (Sugita et al., 1998) and ***ADGRL2*** (***LPHN2***) (Sugita et al., 1998; Ichtchenko et al., 1999) are expressed in the adult human kidney. Analysis of RNA from adult rat kidneys suggests at best a very low level of expression of *Adgrl1* (compared to brain) and *Adgrl2* (compared to brain, lung and liver) (Matsushita et al., 1999).

#### Adgrg1 (Gpr56)

According to the analyzed databases, *Adgrg1* is low expressed in E12.5 mouse ureteric branches (EURExpress, sections 10–13 and 19–22 of the template T60042 in sagittal section) and enriched in the renal cortex of human adult kidneys (Nephroseq). The expression of *ADGRG1* in the adult human kidney has further been supported by published Northern blot (Shashidhar et al., 2005) and western blot data (Huang et al., 2008). Yet, no renal phenotype has been described so far for *Adgrg1* knockout mice (Huang et al., 2008).

#### Adgra2 (Gpr124)

Recently, Calderon-Zamora and co-workers have described that *Adgra2* is expressed on mRNA level (RT-PCR) in the adult kidney of normotensive and hypertensive rats (Calderón-Zamora et al., 2017). A possible role of Adgra2 during kidney development and adult renal physiology is further supported by data from the databases EURExpress (sections 8–10, 16–18) and Nephroseq (enriched in adult renal pelvis).

#### Adgrb1 (Bai1)

Izutsu and coworkers demonstrated by quantitative RT-PCR and immunohistochemistry that *ADGRB1* is expressed on mRNA and protein level in the kidney with prominent expression in proximal and distal tubules (Izutsu et al., 2011).

The databases EURExpress and Nephroseq also suggest a possible role for ***Adgrc2*** (***Celsr2***) in kidney development and physiology (widely expressed, sections 5, 7–10, 17–19; enriched in human adult medulla). In addition, the databases EURExpress and HPA suggest a possible role for ***Adgra3*** (***Gpr125***) (nephrogenic cortex, sections 6–10, 17, 19, 20; low protein expression in adult human tubules).

The Nephroseq database further suggests that ***ADGRE1*** (***EMR1***), ***ADGRB***3 (***BAI3***), and ***ADGRG4*** (***GPR112***) are enriched in the glomerulus, ***ADGRB2*** (***BAI2***) in the renal pelvis, and ***ADGRV1*** (***GPR98***) in the medulla of the adult human kidney. The HPA database indicates that **ADGRG5 (GPR114)** is expressed at low levels in tubules.

Finally, the analysis of aGPCRs during zebrafish development suggests that also ***adgra1l*** (***gpr123l***) (most abundant in kidney and skeletal muscle), ***adgrg3*** (***gpr97***) (strongly expressed during adulthood in most organs), and ***adgrg7b*** (***gpr128b***) (markedly higher expressed in the adult kidney than in any other adult organ or at any other developmental stage) are expressed in the adult zebrafish kidney.

## Conclusion I

Our analysis of publicly available data revealed that the role of aGPCRs during kidney development and physiology is poorly characterized. Yet, a plethora of descriptive data indicates that a large number of aGPCRs are expressed in the kidney from zebrafish to humans in a variety of cell-type specific expression patterns. As most of the available data are based on RNA or “Omics” approaches and as limited information is provided on temporal expression patterns, it is important to validate expression on protein level and to perform detailed spatio-temporal expression analyses. Furthermore, it is fundamental to characterize in detail the available knockout mouse lines for renal phenotypes. Hereby, it should be considered that aGPCRs might have overlapping functions and thus, due to the high number of expressed aGPCR members in the kidney, it might be necessary to utilize double knockout mice (e.g., *Adgrf5*/*Adgrl4*) to elucidate the function of aGPCRs during kidney development and physiology.

## Kidney disease and cancer

Chronic kidney disease (CKD) represents the fastest growing pathology worldwide (Kam Tao Li et al., 2013) with an increase of over 90% from 1990 to 2010 (Lozano et al., 2012). The prevalence of CKD is in many countries >10% (Ortiz et al., 2014; Mills et al., 2015). Independent of the original cause of impaired renal function (e.g., diabetes mellitus, 30–40%; hypertension, 20%; inflammation of the glomeruli (glomerulonephritis), 13%; interstitial nephritis, 10%; polycystic kidney disease, 6%) the common final outcome of almost all progressive CKDs is renal fibrosis (Liu, 2011; Humphreys, 2017). CKDs represent an important risk factor for cardiovascular or cerebrovascular diseases and progress toward end-stage renal disease (ESRD) (Eckardt et al., 2013; Balogun et al., 2017), which can only be treated by renal replacement therapies (RRT) like hemodialysis, peritoneal dialysis, or transplantation (Eckardt et al., 2013). Yet, despite improvements in immunosuppressive medications and renal allograft survival, there is an increasing shortage of available donor kidneys. For example, from 2003 to 2013, the number of patients waiting for a kidney transplant has doubled in the United States. This is mainly due to the fact that diabetes, historically the most common cause of ESRD, is now the fastest rising cause of ESRD (McCullough et al., 2009; Matas et al., 2015). The standard therapy of CKD is antiproteinuric treatment mostly by blockade of the renin-angiotensin system and the management of blood pressure as well as blood glucose levels. This therapeutic regimen slows the progression of the disease, but it cannot reverse or functionally repair the damage, or at least stop progression. In addition, the effect of this regimen depends significantly on the stage of CKDs. In recent years, therefore, new strategies have been proposed for the treatment of CKD that focus on several factors that have been linked to the progression of kidney disease. The main focus is on drugs that have anti-inflammatory and anti-fibrotic effects. At present, these drugs are being tested with the aim of preserving kidney function (Pena-Polanco and Fried, 2016).

Besides CKD, kidney cancer represents a significant socio-economic challenge. Every year around 64,000 patients in the United States and approximately 115,000 patients in Europe are diagnosed with kidney cancer representing 5% of all new diagnosed cancers. In addition, kidney cancer causes yearly in the United States and in Europe nearly 15,000 and 49,000 deaths, respectively (Siegel et al., 2017). The most common kidney tumor is the renal cell carcinoma (RCC) comprising 80% to 90% of all malignant kidney tumors and 70–80% of all solid renal tumors (Ljungberg et al., 2011; Ridge et al., 2014). It originates from the epithelial tubular cells. RCC is characterized by an asymptomatic disease course and thus is often diagnosed late explaining the high mortality rates. It needs to be noted that during the last years it has been recognized that renal cell tumors are more heterogeneous on a histologically and molecularly level than thought. Consequently, the World Health Organization (WHO) classification of renal cell tumors has just recently been modified (Inamura, 2017). The major current subtypes are clear cell RCC (ccRCC, proximal tubule origin), papillary RCC (pRCC, proximal tubule origin), and chromophobe RCC (chRCC, collecting duct origin). ccRCC is a hypervascularized (fragile thin-walled, staghorn-shaped vasculature). Possible causes of ccRCC are mutations in the *VHL* gene (90%) as well as genetic aberrations of mTOR pathway proteins (Low et al., 2016; Inamura, 2017; Shingarev and Jaimes, 2017). Type 1 pRCC is characterized by MET proto-oncogene-activating mutations and type 2 with activation of the NRF2/antioxidant response element pathway due to the increased oxidative stress. In contrast to ccRCC, pRCC is characterized as hypovascularized region compared to the adjacent parenchyma (Herts et al., 2002). chRCC is a rare cancer and the least aggressive of all RCC subtypes. It is characterized unlike ccRCC by thick-walled blood vessels (Low et al., 2016; Inamura, 2017; Shingarev and Jaimes, 2017). Finally, ccRCC shows a less favorable outcome compared with pRCC and chRCC and has a greater propensity to metastasize (Low et al., 2016; Inamura, 2017; Shingarev and Jaimes, 2017).

Complete surgical tumor resection (radical or partial nephrectomies) remains the standard of care for all localized RCCs. Resection of smaller solitary tumors offers a cure for most patients (Autorino et al., 2017). However, this procedure often results in nephron loss as well as a decreased glomerular filtration rate in CKD stage 3 or higher associated with cardiovascular complications, including death (Weight et al., 2010). In the case of unresectable or metastatic RCC, a systemic immunotherapy (IL-2, severe toxicity) or systemic treatment with antiangiogenic factors (frequently leads to hypertension and proteinuria) or mTOR inhibitors is required (Sánchez-Gastaldo et al., 2017; Shingarev and Jaimes, 2017). It should be noted that a large variety of cancers as well as different cancer therapies cause by themselves acute kidney injury and/or CKD through several mechanisms (Małyszko et al., 2017).

Collectively, kidney diseases including kidney cancer represent a major socio-economic burden for which no effective therapy is available. This illustrates the importance to identify early markers of RCC and to better understand on a molecular basis CKD as well as the regenerative potential of the kidney to elucidate new therapeutic targets to delay late stage CKD progression, to stop CKD progression or in the best case to reverse kidney injury.

### Relevance of aGPCRs in cancer

The GPCR superfamily is currently responsible for the therapeutic effect of 33% of all drugs approved by the U. S. Food and Drug Administration (FDA) (Santos et al., 2017). Thus, GPCRs represent the most successful class of pharmaceutical targets. While only a handful of GPCRs have been demonstrated to be oncology drugs, GPCRs play key roles in cancer and thus pharmacological modulation of GPCR function has great potential for the development of novel anti-cancer therapeutics (Nieto Gutierrez and McDonald, 2017). Notably, there is also published evidence for the role of aGPCRs in cancer suggesting them as attractive drug targets. For example, increased expression levels of *ADGRG1, ADGRE5*, and *ADGRF5* has been detected in a variety of human cancers such as glioma (*ADGRG1*) (Shashidhar et al., 2005), gastric (Liu et al., 2005), pancreatic (He et al., 2015), esophageal (Aust et al., 2002), prostate (Ward et al., 2011), colorectal carcinomas (Steinert et al., 2002) (*ADGRE5*), and breast cancer (*ADGRF5*) (Tang et al., 2013). Moreover, their importance in tumorigenesis has been substantiated in xenograft tumor models. *ADGRG1* knockdown resulted in melanoma growth inhibition and regression (Ke et al., 2007), while knockdown of the other 2 aGPCRs reduced lung (*ADGRF5*) (Tang et al., 2013) and/or bone metastasis (*ADGRE5, ADGRF5*) (Ward et al., 2011; Tang et al., 2013). Detailed *in vitro* and *in vivo* analyses further indicated that *ADGRG1* is a prosurvival factor in cancer cells and promotes anchorage-independent growth (Ke et al., 2007) whereas *ADGRE5* (Ward et al., 2011) and *ADGRF5* (Tang et al., 2013) regulate RhoA-dependent cell migration and invasion.

## Relevance of aGPCRs in kidney disease including kidney cancer

### Literature-based evidence supported by database information

As detailed below, our literature search identified publications describing five aGPCRs correlated with kidney disease including renal cancer: *Adgrb1*, ***Adgrg2*** (***Gpr64***), *Adgrl4, Adgrg3*, and *Adgrf5*.

### Adgrb1 suppresses RCC angiogenesis

It is well known that new vascular formation (angiogenesis) is required for tumor growth, invasion and metastasis (Carmeliet and Jain, 2000). This process is directly promoted by VEGF (Carmeliet, 2005). Yet, there are also a large variety of other factors that control tumor angiogenesis including *Adgrb1* (Mathema and Na-Bangchang, 2017). Izutsu and coworkers demonstrated by quantitative RT-PCR a 4-fold decrease in *ADGRB1* expression in RCC tissue compared to normal kidney tissue, whereby *ADGRB1* mRNA and protein expression were lower in advanced RCC than in localized RCC tissues. Moreover, the authors identified a negative correlation between microvessel density and *ADGRB1* protein expression. VEGF expression was not affected (Izutsu et al., 2011). These data suggested that downregulation of *ADGRB1* during RCC contributes to an improved tumor angiogenesis and thus enhanced tumor growth. This is in agreement with the previous findings of Kudo and coworkers who studied the potential of *ADGRB1* as an anti-angiogenic factor in RCC based on the known inverse correlation of its expression with vascular density in other tumor types (Kudo et al., 2007). They demonstrated in an *in vivo* tumor model (subcutaneous inoculation of the RCC cell line Renca) that *ADGRB1* overexpression in Renca reduced tumor growth due to reduced microvessel density which was associated with reduced VEGF expression levels (Kudo et al., 2007). The role of *ADGRB1* is further supported by the FireBrowse database which indicates that *ADGRB1* is downregulated in chRCC (0.010-fold, Table 2) and the cBio database indicating a mutation rate of 11% in chRCC. The observed downregulation of *ADGRB1* in chRCC might be an explanation for an increased vascularization associated with chRCC (Low et al., 2016; Inamura, 2017; Shingarev and Jaimes, 2017) and is consistent with no change in hypovascularized pRCC (Herts et al., 2002). The lack of downregulation in hypervascularized ccRCC might be due to the different types of vessels (fragile vs. thick-walled; Low et al., 2016; Inamura, 2017; Shingarev and Jaimes, 2017).

### Adgrg2 is involved in tumor invasiveness

*ADGRG2* is highly expressed in several human cancer cell lines including 8 human RCC cell lines: CAKI-1, 786-0, A498, ACHN, SN12-C, (all ccRCC), TK-10, RXF 393, and UO-31 (Richter et al., 2013). In addition, it is overexpressed in Ewing's sarcoma cell lines (Richter et al., 2013). Ewing's sarcoma is an aggressive, small round-cell tumor with a strong potential to metastasize. Typically it arises from the trunk and long bones of children or young adults (Toomey et al., 2010). Yet, it can also primarily occur in soft tissues, like the kidney (extra-skeletal Ewing's sarcomas), whereby its cellular origin is still unknown (Faizan et al., 2014). Richter and coworkers demonstrated that inhibition of *Adgrg2* expression impaired colony formation *in vitro* (A673, SB-KMS-KS1 and TC-71) and suppressed local tumor growth and metastasis in an immunodeficient mouse xenograft model of human cancer [SB-KMS-KS1/Rag2^(−/−)^γC^(−/−)^]. Finally, the authors present evidence that *Adgrg2* regulates invasiveness and metastatic spread via induction of placental growth factor (PGF) and matrix metalloproteinase 1 (*Mmp-1*) expression (Richter et al., 2013). Consequently, the FireBrowse database shows that *ADGRG2* is only upregulated in ccRCC (2.34, Table 2), the most metastatic RCC subtype (Low et al., 2016; Inamura, 2017; Shingarev and Jaimes, 2017).

In addition, Nephroseq data suggest that *ADGRG2* is upregulated in diabetic nephropathy (human, mouse model), CKD (human) as well as in the mouse Berthier model of lupus nephritis with proteinuria. This finding is in agreement with the fact that these diseases as well as RCC are associated with abnormal expression levels of MMPs (Nakamura et al., 2000; Catania et al., 2007; Jiang et al., 2010), including MMP-1 (Nakamura et al., 1993, 2000; Bhuvarahamurthy et al., 2006) which is known to be regulated by *Adgrg2* (Richter et al., 2013). In addition, it has been demonstrated that, despite the large number of MMPs known to be expressed in the healthy kidney (Catania et al., 2007), changes in a single MMP can cause kidney disease (Cheng et al., 2006). Moreover, it has been shown that diabetes and systemic lupus erythematosus can cause CKD through excessive levels of blood glucose or deposition of immune complexes, respectively, resulting in defects in the basement membrane, podocyte effacement, increase in mesangial cell number and extracellular matrix (Fibbe and Rabelink, 2017; Sharaf El Din et al., 2017). Ultimately, these conditions lead to scarring and stiffening of the glomerulus (glomerulosclerosis), tubulointerstitial fibrosis and CKD (Meguid El Nahas and Bello, 2005).

### Adgrl4 correlates with better prognosis in ccRCC

As described above in the section about kidney development, *Adgrf5*/*Adgrl4* double knockout mice exhibit significantly reduced kidney function. Around half of the double-deficient mice died perinatally, while the other half showed growth impairment and all died within 3 weeks to 3 months postnatally. The analysis of 7 weeks-old mice revealed massive proteinuria and uremia. Analyses of 4 weeks-old mice revealed a set of pathological features indicating glomerular thrombotic microangiopathy: schistocytes in the subendothelial zone of the glomerular capillaries, loss of endothelial fenestration and fusion of podocytes foot processes (Lu et al., 2017). Notably, even though *Adgrl4 and Adgrf5* are highly expressed in endothelial cells, endothelial-specific deletion of *Adgrf5*/*Adgrl4* utilizing *VE-Cad-Cre* mice resulted in no renal phenotype. This suggests that at least one of these aGPCRs might have a function in a renal cell type.

A possible role of *ADGRL4* in renal cancer has been proposed by Masiero and collaborators. They analyzed the expression profile of 1,080 primary human cancer samples including 170 ccRCC samples (Masiero et al., 2013). Utilizing a validated anti-ADRGL4 antibody they detected ADGRL4 expression in the endothelial cells and pericytes of all investigated normal and pathological tissues. In ccRCC samples, ADGRL4 expression was significantly higher than in the healthy control and its expression level correlated positively with ccRCC microvessel density. Importantly, the authors demonstrated that ADGRL4 is required for proper endothelial sprouting and vessel formation *in vitro* and *in vivo* and that its inhibition drastically reduced tumor growth improving survival (Masiero et al., 2013). FireBrowse data confirm this observation indicating an upregulation (2.13-fold, Table 2) only in the hypervascularized ccRCC subtype (Low et al., 2016; Inamura, 2017; Shingarev and Jaimes, 2017). It should be noted that *ADGRL4* is downregulated (0.179-fold) and mutated (11%) in chRCC (Table 2), which might be explained, similarly as for *ADGRB1*, by the different types of vessels (fragile vs. thick-walled; Low et al., 2016; Inamura, 2017; Shingarev and Jaimes, 2017).

Besides a role in kidney, *ADGRL4* upregulation is according to the Neprhoseq database associated with IgA nephropathy (33 human samples, 2.757 fold change, *p* = 5.18E-12) as well as lupus nephritis (46 human samples, 5.493 fold change, *p* = 2.46E-7). In addition, it has been associated with diabetic nephropathy in mice (39 samples, 1.525 fold change, *p* = 0.002) but not in humans (22 samples,−2.116 fold change, *p* = 0.999). Diabetic nephropathy, IgA nephropathy and lupus nephritis are all glomerular diseases in which sclerosis, increases in the mesangial matrix and hypercellularity of the mesangial cells are common pathological features (Meguid El Nahas and Bello, 2005; Fibbe and Rabelink, 2017; Sharaf El Din et al., 2017). Yet, while *Adgrl4* is clearly associated to endothelial cells and the microvasculature, there appears no common phenotype in regards to an increased microvascular density (as in hypervascularized ccRCC). This suggests, similar as the lack of a renal phenotype in conditional *Adgrf5*/*Adgrl4 x VE-Cad-Cre* mice (see above), that *Adgrl4* has a so far unknown function in a non-endothelial type. It should be once more noted that according to EURExpress (sections 5–8, 14–17) *Adgrl4* is detected in the developing metanephros of E14.5 mice. Yet, based on these data it is difficult to determine if *Adgrl4* is expressed in cells other than the endothelial cells of the microvasculature.

### Adgrg3 and its relation with diabetic nephropathy

*Adgrg3* is highly expressed in immune cells, but its function remains largely unknown (Peng et al., 2011). Consistent with the fact that the kidney develops macrophage-associated local inflammation in response to metabolic disorders, such as insulin resistance and diabetes (Navarro-González and Mora-Fernandez, 2008; Declèves and Sharma, 2015), *Adgrg3* is according to the Nephroseq database upregulated in the mouse model of diabetic nephropathy (12 samples, 2.938 fold change, *p* = 2.55E-6). Loss of function experiments revealed that *Adgrg3* has a crucial role in maintaining normal splenic architecture and regulating B cell development (Wang et al., 2013). Notably, Shi and coworkers reported recently that more (2-fold) macrophages invaded the kidney of *Adgrg3* knockout mice on high-fat diet (HFD) compared to HFD wildtype mice (Shi et al., 2016). This phenotype was correlated to higher expression levels of the inflammatory factor TNF-α. Yet, major metabolic phenotyping showed no difference between *Adgrg3* knockout and wildtype mice on HFD.

In RCCs, *ADGRG3* is according to the FireBrowse database upregulated in all three RCC subtype with the highest expression in chRCC (3.89-fold, Table 2). This is consistent with the fact that immune cells and inflammatory processes significantly contribute to RCC development and disease progression (de Vivar Chevez et al., 2014).

### Additional database information

Overall, the data obtained from the utilized databases indicate that almost all aGPCRs are associated to kidney diseases including APOL1-associated kidney disease, ccRCC, chRCC, CKD, diabetic nephropathy, focal segmental glomerulosclerosis (FSGS), hypertension-injured kidney, IgA nephropathy, lupus nephritis, minimal change disease, membranous glomerulopathy, pRCC, and vasculitis-injured kidney. Below we discuss the diseases to which expression changes of several aGPCRs have been associated.

#### CKD

Long-term kidney damage independent of its origin progressively leads to a chronic disease state characterized by an irreversibly low glomerular filtration rate (Meguid El Nahas and Bello, 2005). According to the Nephroseq database, 18 out of 33 human aGPCRs are upregulated in CKD compared with healthy counterparts. Additionally, in two thirds of the cases (12 out of 18), the fold change increase is >3. The altered expression of that many aGPCRs is most likely a reflection of the large variety of etiologies underlying CKD and the multiple common pathophysiological processes involved such as inflammation, ischemia, hypoxia, fibrosis, and metabolic waste accumulation.

#### Lupus nephritis, diabetic nephropathy, IgA nephropathy

These are the kidney diseases that are associated with upregulation of a larger number of aGPCRs. Moreover, they exhibit similar aGPCR signatures which might reflect that all these diseases are glomerular diseases sharing common pathological features such sclerosis, increases in the mesangial matrix and hypercellularity of the mesangial cells (Meguid El Nahas and Bello, 2005; Fibbe and Rabelink, 2017; Sharaf El Din et al., 2017). Increased expression levels were found for ADGRL2, ADGRL4, ADGRE1, ADGRE5, ADGRG2, and ADGRG6 in lupus nephritis and diabetic nephropathy, whereby ADGRL2, ADGRL4 and ADGRE5 were also upregulated in IgA nephropathy (Table 2). As described above, altered expression of *Adgrg2* reflects the involvement of MMPs in these diseases (Nakamura et al., 1993, 2000; Bhuvarahamurthy et al., 2006; Catania et al., 2007; Jiang et al., 2010; Richter et al., 2013). Altered expression of ADGRE1 and ADGRE5 is most likely due to inflammatory processes underlying the progression of the three diseases (Galkina and Ley, 2006; Imig and Ryan, 2013). Adgre1 is in mice a macrophage marker (Lin et al., 2010) and in humans a marker of eosinophilic granulocytes (Hamann et al., 2007). Adgre5 is widely expressed along the lymphoid and myeloid lineages (Hamann et al., 2015). Consequently, *Adgre5* is upregulated in autoimmune diseases (Wandel et al., 2012; Lin et al., 2017). In contrast, the role of *ADGRL4* in these diseases remains unclear as discussed above. In regards to *ADGRL2* and *ADGRG6* no published information is available that would allow to conclude what role they might play. *Adgrl2* has previously been associated with EMT in chicken valve heart development (Doyle et al., 2006) and *Adgrg6* with heart trabeculation and myelination (Patra et al., 2014).

#### RCCs

According to the here analyzed databases aGPCRs which are related to vascularization (*Adgre1, Adgre5, Adgra2*) (Wang et al., 2005; Cullen et al., 2011; Wandel et al., 2012) or inflammatory processes (*Adgre1, Adgre2, Adgre5*) (Gray et al., 1996; Taylor et al., 2005; Hamann et al., 2007; Kuan-Yu et al., 2017) are >2.5-fold upregulated in ccRCC (Table 2). This reflects the high degree of vascularization of ccRCCs (Prasad et al., 2006) and the role of inflammatory related gene expression in its progression (Tan et al., 2011). A role of these genes in vascular and/or inflammation-mediated kidney diseases is further substantiated by the associations with diabetes nephritis (*ADGRE1, ADGRE5*), lupus nephritis (*ADGRE1, ADGRE2, ADGRE5*), renal vasculitis (*ADGRE2, ADGRE5*), IgA nephropathy (*ADGRE5*) and renal hypertension (*ADGRE5*). Furthermore, the hypoxia associated aGPCRs *ADGRB3* (Kee et al., 2004) and *ADGRD1* (Bayin et al., 2016) were >2.5-fold downregulated. In addition, the aGPCRs *ADGRA1, ADGRF1, ADGRF3*, and *ADGRV1* were downregulated for which currently no role can be hypothesized.

In contrast to ccRCC, for chRCC it has been found that the majority of affected aGPCRs (>2.5-fold change) are downregulated (12 out of 15, Table 2). The major known differences between ccRCC and chRCC ist that chRCC is less metastatic, less vascularized and exhibits instead of fragile vessels more thick-walled vessels. Consequently, proangiogenic aGPCRs were not upregulated. In addition, expression of *ADGRG1*, which promotes cell adhesion (Hamann et al., 2015), is strongly upregulated and might explain the lower metastatic character of chRCC. Yet, the pro-migratory aGPCR *ADGRG3* is highly expressed. Collectively, it appears impossible to extrapolate the role of the affected aGPCRs in chRCC without more detailed studies. The same is true for pRCCs, in which 10 aGPCRs were >2.5-fold down- and 5 aGPCRs upregulated. Consistent with its hypoplastic characteristic, the proangiogenic *ADGRL4* was downregulated. Yet, the pro-angiogenic factors *ADGRE1* and *ADGRE5* were upregulated. Note, the FireBrowse data have to be dealt with carefully as no *p*-values are provided.

Overall, it is apparent that in RCCs the majority of aGPCRs is downregulated (28 out of 40), which is in agreement with the described cellular functions of aGPCRs, which for example mediate cell adhesion and directed cell division (Hamann et al., 2015); processes often misregulated in RCCs. Among the upregulated a GPCRs are consequently aGPCRs that promote angiogenesis (*ADGRE1, ADGRE5, ADGRA2*) and inflammation (*ADGRE1, ADGRE2, ADGRE5*) or act in contrast to most other aGPCRs as anti-adhesive molecules (*ADGRG1*). It has for example been shown that ADGRG1 activation promotes melanoma cell migration (Chiang et al., 2017) and is required for proper β-cell proliferation (Dunér et al., 2016). Yet, it is noteworthy that the cellular functions of aGPCRs are still poorly characterized and thus the kidney might provide a chance to better understand the distinct roles of aGPCRs.

## Conclusion II

Our analysis of publicly available data revealed that the role of aGPCRs is also poorly characterized in kidney disease including cancer. However, while the data in the literature is sparse and not always in agreement with available “Omics” data, the publicly available data support the conclusion that aGPCRs are involved in the pathogenesis of a variety kidney diseases. Yet, it needs to be clarified if the majority of the listed associations are due to alterations in vascularization or inflammation of the diseased kidney or if aGPCRs play additional underappreciated kidney-specific roles.

## Future directions

Our analysis indicates that a large number of aGPCRs is associated to kidney development and a variety of kidney diseases suggesting an underappreciated role of aGPCRs in kidney development, physiology and pathophysiology. Due to the limited spatio-temporal expression data for aGPCRs in the kidney it is difficult to conclude if certain aGPCRs are *de novo* expressed during a disease or whether it is a question of reactivation, for example in the context of a fetal gene program. Thus, it is important to generate adequate tools (e.g., validated antibodies and reporter lines) to better characterize the expression patterns of aGPCRs. Considering that multiple single aGPCR knockout mouse lines exhibit no obvious phenotype, further analysis might provide information regarding complementary aGPCRs with compensatory functions. However, it should also be considered that the lack of phenotypes might be due to the lack of a vigorous investigation (e.g., kidney not analyzed, kidney function not challenged) or to an incomplete gene deletion. For example it has been shown that the N-terminal and C-terminal fragment of aGPCRs can have independent functions (Patra et al., 2013). In addition, genetic manipulations of an aGPCR might not affect alternative splice forms (Bjarnadóttir et al., 2007) or induce expression of artificial alternative splice forms. Cell-type specific expression analysis will also help to determine if aGPCRs have a primary role or whether their association is rather due to bystander effects. This, however, appears rather unlikely as aGPCRs regulate a variety of cellular processes known to be essential to kidney development, physiology and pathophysiology. Collectively, it is important that experts in kidney development and disease collaborate with experts in the field of aGPCRs to elucidate the role of aGPCRs in kidney development, physiology and pathophysiology in order to determine if this knowledge can be utilized for better diagnosis and/ or treatment of kidney diseases including RCCs.

## Author contributions

SC-V and FE performed literature as well as database review and analysis and wrote the manuscript.

### Conflict of interest statement

The authors declare that the research was conducted in the absence of any commercial or financial relationships that could be construed as a potential conflict of interest.
